# A Mini-System Integrated with Metal-Oxide-Semiconductor Sensor and Micro-Packed Gas Chromatographic Column

**DOI:** 10.3390/mi9080408

**Published:** 2018-08-17

**Authors:** Jianhai Sun, Zhaoxin Geng, Ning Xue, Chunxiu Liu, Tianjun Ma

**Affiliations:** 1 State Key Laboratory of Transducer Technology, Institute of Electronics, Chinese Academy of Sciences, Beijing 100190, China; cxliu@mail.ie.ac.cn (C.L.); mmmmtj@126.com (T.M.); 2 School of Science, Minzu University of China, Beijing 100081, China; zxgeng@semi.ac.cn

**Keywords:** packed gas chromatographic column, metal-oxide-semiconductor array sensor, sensitive material, environmental monitoring

## Abstract

In this work, a mini monitoring system integrated with a microfabricated metal oxide array sensor and a micro packed gas chromatographic (GC) column was developed for monitoring environmental gases. The microfabricated packed GC column with a 1.6 m length was used to separate the environmental gas, and the metal oxide semiconductor (MOS) array sensor, fabricated with nano-sized SnO-SnO_2_ sensitive materials, was able to effectively detect each component separated by GC column. The results demonstrate that the monitoring system can detect environmental gas with high precision.

## 1. Introduction

With the development of industry, factories and automobiles are crowded in cities, making the city’s environment full of harmful gases, such as CO, benzene, SO_2_, and others, which are especially damaging to human health [1,2]. Moreover, the harmful components mixed in the air are very complicated. For the purpose of air filtering or air quality monitoring, these harmful components need to be accurately identified and monitored so that these harmful components can be effectively removed from the air or real-time detected. Therefore, the demand on gas sensors with high performance is very urgent.

MOS (metal oxide semiconductor) sensors which are surface-modified with different sensitive materials can detect different gases [3,4,5,6,7,8,9,10,11,12,13,14,15,16,17,18,19], such as carbon monoxide, sulfur dioxide, hydrogen sulfide, ammonia, and so on. Therefore, MOS sensors have become important environmental gas detectors and have attracted the attention of many researchers. In recent years, a large number of research papers on various MOS detectors have been reported. Nicoletti S and other research teams [4,5,6,7,8,9,10,11,12,13,14,15] have developed MOS gas detectors using nanocomposites as sensitive materials. Tomer V and other research teams [16,17] demonstrated MOS sensors with high performance that were doped with Ag or other catalytic materials. Kim [18] and other groups [19,20,21,22,23,24] also have successfully developed a variety of MOS sensors using sensitive materials based on binary or multiple metal oxides which have better reactivity towards target gases than single oxides. In addition, some research groups have proposed some other methods to improve selectivity and sensitivity of MOS sensors [25,26,27,28].

MOS sensors can detect environmental gas with high sensitivity, however, MOS sensors have poor resolution, and the problem of cross-talk interference between gases with similar properties is very serious. For example, when NO is detected, NO_2_ will interfere with it. Similarly, SO_2_ will also interfere with the detection of H_2_S. Chromatography is a powerful analytical technique, which is able to separate mixed gases, as the retention time of gases in the stationary phase is different. Then the completely separated gases were quantified by high sensitive detectors. This method avoids the cross-talk interference between gases, thus greatly improving detection precision. In recent years, miniaturized gas chromatography columns (GCCs) have received increased attention and are under development in many laboratories [29,30,31,32].

Therefore, in this work, a microfabricated metal oxide array sensors based on nanosized SnO-SnO_2_ sensitive materials were fabricated. Compared to pure SnO_2_ sensitive materials, the SnO_2_ sensitive material doped with Sn^2+^ has more oxygen vacancies, which has a property of N type semiconductors and higher gas activity. Moreover, in order to solve the cross-talk interference between gases, in this work, a micro GC column, which has powerful separation ability, was proposed to separate analytical sample. After these interfering gas components were separated by the GC column, they were able to be detected with high precision by the MOS detector.

## 2. Materials and Methods

### 2.1. Materials and Reagents

In this work, in order to evaluate sensitivity of the fabricated MOS array sensor, carcinogenic gas, benzene (Sample I, purchased from Beijing Hua Yuan Gas Chemical Industry Co., Ltd., Beijing, China) was used as the test target; the concentration of benzene is 5 ppm. Sample II (Beijing Hua Yuan Gas Chemical Industry Co., Ltd., Beijing, China) was composed of 3 compounds (benzene, CO, and SO_2_, the concentrations are 5 ppm, 500 ppm, and 505 ppm, respectively). Porapak Q with a diameter of 100 μm was purchased from Sigma-Aldrich (St. Louis, MO, USA). 

### 2.2. Experimental Setup

**Figure 1:**
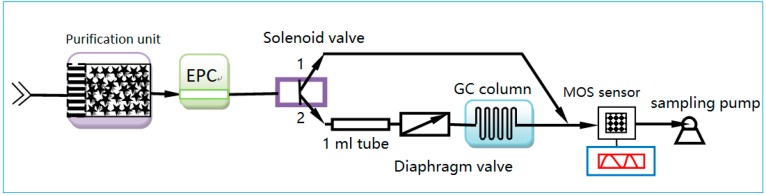
Configuration of the mini monitoring system.

**Figure 2:**
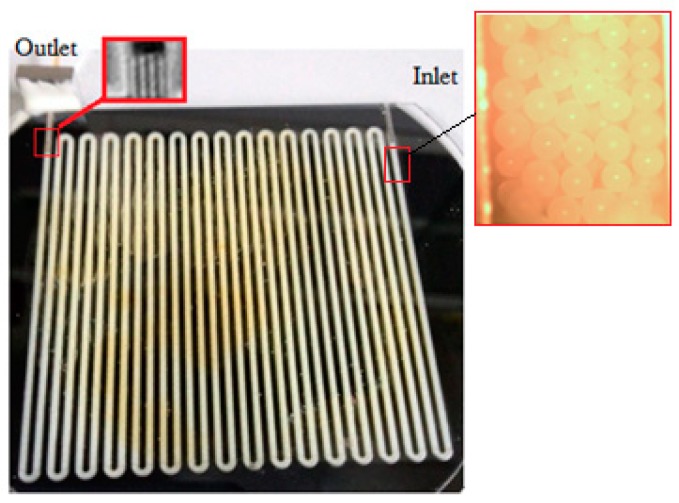
The fabricated micro GC column packed with porapak Q stationary phase.

In this work, the mini system integrated with a micro GC column and micro MOS array sensor was proposed for monitoring environmental harmful gas. The sample injected by a sampling pump was transported into the packed column through a valve purchased from Valco Instruments Company Incorporated (VICI). Clean air was used as carrier gas, the flow rate of which was controlled by electronic pressure control (EPC) technique. The setup of the system is shown in Figure 1. The working principle of the system is as follows. Firstly, a certain amount of sample was collected by sampling pump, and the sample was directly transported into the detector through port 1 of the solenoid valve, which measured the total amount of gas in the sample. Then, a certain amount of gas was collected and transported into the tube through port 2 of the solenoid valve when the diaphragm valve is in a closed state. Finally, the sampling probe was detached from the pollutant source, and fresh air, which acted as carrier gas, was pumped and transported through the sample into the micro GC column by opening the diaphragm valve. Then these components were separated by the micro GC column and detected by the MOS sensor. In this work, a micro GC column with length of 1.6 m [32] was used for separating polluted gases is a packed column developed by our lab. Channels of the packed GC column were fabricated using a laser etching technology (LET) which can easily fabricate deep well-shaped channels on glass wafer or silicon wafer, and the fabricated column (refer to Figure 2) with a rectangular cross section of 1.2 mm (depth) × 0.6 mm (width) has a large aspect ratio of 2:1. In order to effectively separate the polluted gas (such as CO, SO_2_, and benzene, etc.), Porapak Q, acting as stationary phase, was packed in the column, and the packing process is detailed as follows. First, the inlet of the column was emerged into the porapak Q powder and the outlet was connected with a pump (the flow rate of the pump needs to be in the range of 1–5 L/min). Then, the porapak Q powder was transported into the micro channels under the pumping action. In order to uniformly pack the column, the column needs to be gently beaten during the packing process.

### 2.3. Fabrication of MOS Array Sensor

It is well known that the MOS array sensor consist of a SnO-SnO_2_ nano-metric film was deposited over a silicon micro-machined substrate implemented with platinum heater and two electrodes for contacting thin sensitive element. The MOS array sensor comprising of four completely independent detectors was fabricated based on micro-electro-mechanical system (MEMS) technology, and silicon was used as substrate wafers. The fabrication process of the chip was accomplished as follows. (1) A layer of boron ions (B^+^) with thickness of 5 µm was implanted into the surface of silicon as a mask for the corrosion of silicon and a supported beam for hotplate and electrodes. The concentration of B^+^ was 1 × 10^19^ cm^−3^, which was high enough for the self-stop corrosion. (2) A layer of SiN film with thickness of 300 nm was deposited on silicon wafer using low pressure chemical vapor deposition (LPCVD) technology. (3) The hotplate was fabricated as a 20 nm/150 nm Cr/Pt stack deposited by the magnetron sputtering technology and patterned by the lift-off technology. (4) A layer of SiN film with thickness of 200 nm acted as passivation layer (to provide electrical insulation between the platinum heater and the sensing layer) was deposited on the top of the Pt hotplate using plasma enhanced chemical vapor deposition (PECVD) technology. (5) The electrodes were realized as a 30 nm/200 nm Cr/Au stack deposited by the magnetron sputtering technology and patterned by the lift-off technology.

**Figure 3:**
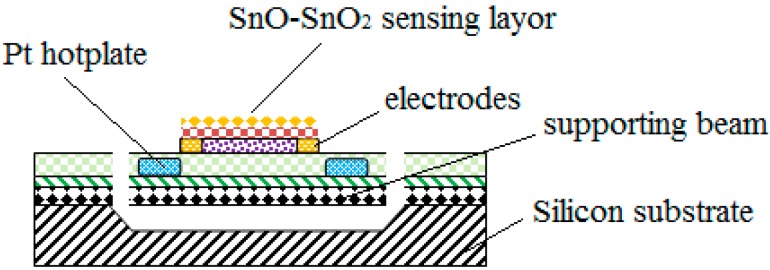
A schematic representation of the whole structure.

SnO_2_ is a direct broadband gap semiconductor material. SnO_2_ has a wide bandgap; a pure and ideal chemical ratio SnO_2_ has high resistance. However, after the SnO_2_ material was doped with SnO, the SnO_2_ material deviates from its ideal chemical ratio, which makes the lattice with mixed normal ions (Sn^2+^, Sn^4+^) and oxygen negative ions (O^2−^) in the absence of state, easily producing oxygen vacancy. As oxygen vacancies are able to form two donor levels in the forbidden band, and the two donor levels formed by oxygen vacancies have been completely dissociated at room temperature, SnO_2_ has the property of N type semiconductors. The N type SnO_2_ thin film has excellent performance. Its carrier concentration can reach 10^19^–10^21^ cm^−3^, and its conductivity can reach 10^−3^–10^−2^ Ω·cm. Therefore, in this work, in order to improve the activity of SnO_2_ sensitive material, SnO materials are doped into SnO_2_ sensitive materials according to a certain mass ratio. The sensitive film was fabricated and the process was defined as follows, and a schematic representation of the whole structure is depicted in the Figure 3.

**Figure 4:**
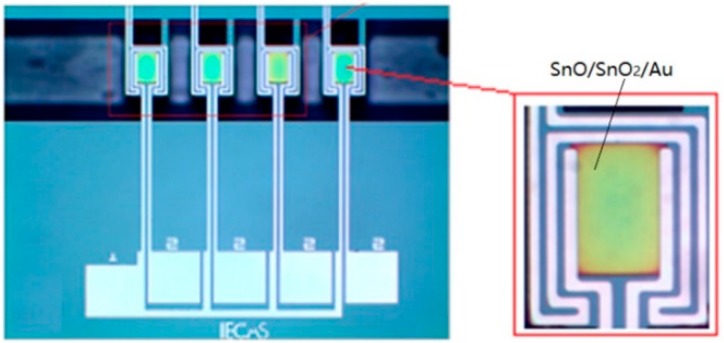
Photo of the MOS array sensor.

First, over the surface of Au electrodes (for contacting thin sensitive film), a layer of SnO and SnO_2_ thin film deposited over the hotplate surface have been carried out by sputtering technology, and the thickness of the SnO and SnO_2_ thin film were 50 nm and 150 nm, respectively. In order to increase the selectivity and sensitivity of the sensitive film, an extremely thin Au film acting as catalytic material with thickness of 5 nm was deposited over its surface. Noble metals [33] Au, as a surface active center, has the function of catalytic oxidation. In addition, the noble metal Au has large electronic affinity, which can accelerate the transfer of electrons from semiconductors to a noble metal and improve the sensitivity. Finally, the release process of the supporting beam was shown as follows. A layer of photoresist with thickness of 2 µm was coated and patterned as an etch mask for silicon nitride. After the two layers of silicon nitride were etched by reactive-ion etching (RIE) technology, a deep reactive-ion etching (DRIE) process was utilized to remove the diffusion of silicon in the micro channels. Then the supporting beam was released through a silicon etch (using 40% wt% KOH solution at 80 °C for 70 min). The width and length of chip (refer to Figure 4) was 8 mm and 10 mm, respectively, and the active area of the sensor (for each of the four sensors) was only 1 × 4 mm^2^, consisting of a platinum resistor acting as heater.

## 3. Results

### 3.1. Gas Sensing Characteristics of the MOS Array Sensors

In order to evaluate sensitivity of the fabricated MOS array sensor based on SnO-SnO_2_ sensitive material, a MOS sensor coated with pure SnO_2_ as a sensitive material was used for a comparison experiment. Table 1 shows the response characteristics of the two sensors to same samples (sample II). From the response amplitude of the output, the sensitivity of the MOS sensor doped with SnO is much greater than that of the MOS sensor coated with pure SnO_2_ only.

**Figure 5:**
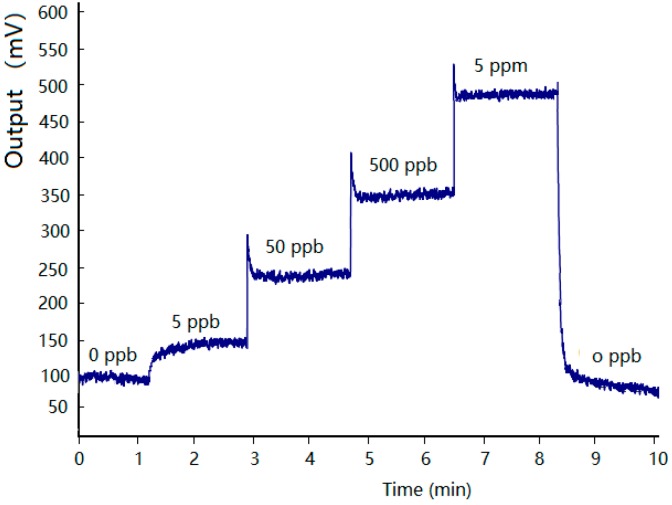
Response of the fabricated sensor to benzene at different concentrations.

The concentration response characteristics of the MOS array sensor fabricated with a nano-sized SnO-SnO_2_ sensitive material were analyzed and evaluated. Samples containing benzene were diluted to different concentrations and successively transported directly to the MOS sensor without passing through the micro GC column in periodically. Figure 5 shows the response curve of the fabricated sensor. As we can see that the detector can obtain an obvious response to extremely low concentrations of gas, moreover, the sensor has a response gradient to different concentration gases, and there is a linear relation between the increase of output signal and the concentration.

### 3.2. Rapid Detection of Polluted Gases

**Figure 6:**
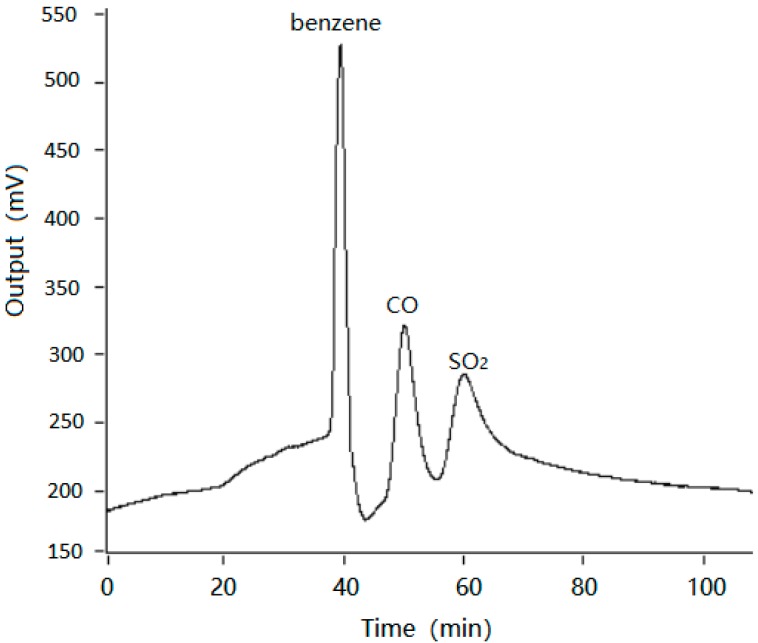
Chromatogram of benzene, CO, and SO_2_.

To evaluate the performance of the fabricated monitoring system integrated with micro GC column and micro MOS array sensor. The experiment was carried out with sample II at a flow rate of 10 mL/min, and the inlet pressure of column was 80 psi and temperature of the column was 80 °C. As can be seen from the chromatogram (refer to Figure 6), the MOS array sensor integrated with chromatographic column was able to detect each component without mutual interference, solving the technical bottleneck of cross-talk interference between gases. That is to say, this detection method makes good use of the function of chromatography to solve the inherent technical bottleneck of MOS sensor. The specific concentration of each component can be accurately defined by comparing the area of each chromatographic peak with the corresponding peak area of the known concentration. Therefore, the high sensitive detector can overcome its own defects by integrating chromatography, which will greatly expand its applications and play an important role in environmental monitoring.

## 4. Conclusions

The work here demonstrated that it was possible to fabricate a mini monitoring system integrated with a MOS array sensor and a micro packed chromatographic column. By using a powerful chromatographic separation capability, the MOS array sensor was able to detect each component with high resolution and solve the technical bottleneck of mutual interference between gases. Therefore, the fabricated MOS array detector with high sensitivity can overcome its own defects by combining chromatographic techniques, which will greatly expand its applications and play an important role in environmental monitoring.

## Figures and Tables

**Table 1:** The average response of the two sensors to same sample.

Average Response	MOS Sensor (Coated with SnO/SnO_2_)	MOS Sensor (Coated with SnO_2_ Only)
The average response of benzene (mV)	290.0	165.0
The average response of CO (mV)	120.0	75.0
The average response of SO_2_ (mV)	85.0	48.0
